# Generating Membrane Curvature at the Nuclear Pore: A Lipid Point of View

**DOI:** 10.3390/cells11030469

**Published:** 2022-01-29

**Authors:** Bas W. A. Peeters, Alexandra C. A. Piët, Maarten Fornerod

**Affiliations:** Department of Cell Biology, Erasmus MC, Dr. Molewaterplein 40, 3015 GE Rotterdam, The Netherlands; bas.peeters@ndm.ox.ac.uk (B.W.A.P.); a.piet@erasmusmc.nl (A.C.A.P.)

**Keywords:** membrane curvature, nuclear pore complex, nuclear envelope, lipids, SMPD4

## Abstract

In addition to its structural role in enclosing and protecting the genome, the nuclear envelope (NE) forms a highly adaptive communication interface between the cytoplasm and the nuclear interior in eukaryotic cells. The double membrane of the NE is perforated by nuclear pores lined with large multi-protein structures, called nuclear-pore complexes (NPCs), which selectively allow the bi-directional transport of ions and macromolecular cargo. In order to nucleate a pore, the inner and outer nuclear membrane have to fuse at the site of NPC insertion, a process requiring both lipid bilayers to be deformed into highly curved structures. How this curvature is achieved and which factors are involved in inducing and stabilizing membrane curvature at the nuclear pore remain largely unclear. In this review, we will summarize the molecular mechanisms thought to be involved in membrane curvature generation, with a particular emphasis on the role of lipids and lipid metabolism in shaping the nuclear pore membrane.

## 1. Introduction

**Figure 1:**
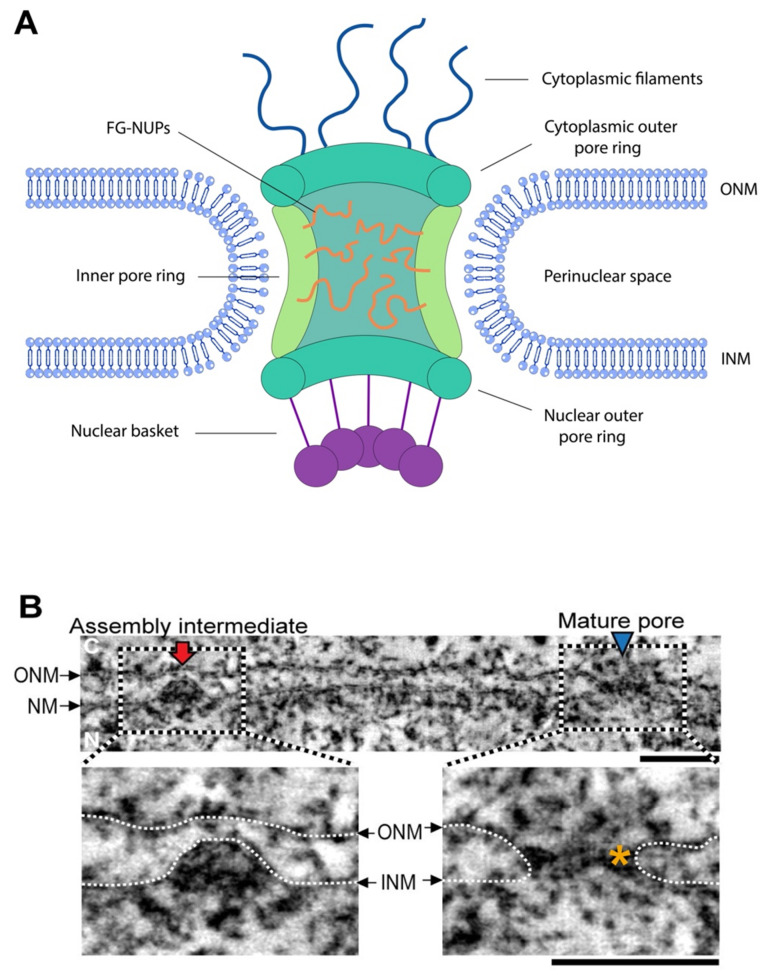
(Ultra)structural characteristics of the nuclear pore and its assembly intermediates (**A**) Schematic representation of the nuclear pore complex (NPC) embedded into the nuclear envelope. (**B**) Electron-tomographic slice of a region of an isolated nuclear envelope (NE) of HeLa cells. An assembly intermediate (i.e., a dome-shaped evagination of the INM) and a mature pore are indicated by a red arrow and a blue arrowhead, respectively. The highly curved pore membrane domain is indicated by an orange asterisk. ONM, outer nuclear membrane; INM, inner nuclear membrane. Scale bar = 100 nm. Reprinted and adapted from [9].

The nuclear envelope (NE) is a crucial organelle of the eukaryotic cell and is one of the iconic features that distinguishes eukaryotic from prokaryotic organisms. It separates the nuclear genome from the protein translation machinery in the cytoplasm, providing eukaryotic cells with an additional tool for the regulation of gene expression compared to their prokaryotic predecessors [1]. The NE is formed by two closely opposed lipid bilayers, the inner nuclear membrane (INM) and outer nuclear membrane (ONM). The latter is in direct continuity with the membranes of the rough endoplasmic reticulum (ER). The two membranes are separated from each other by a narrow gap (~30–50 nm in width), termed the perinuclear space, which connects with the ER lumen. The individual bilayers of the nuclear envelope locally converge and merge into highly curved pore-membrane domains, where nuclear pore complexes (NPCs) insert into the nuclear envelope. NPCs are large multisubunit protein assemblies consisting of nucleoporins (Nups)—with a total estimated molecular mass of ~110 MDa in vertebrates and ~52 MDa in yeast [2,3,4,5,6]—that allow for selective bidirectional transport of macromolecules between the nucleus and the cytoplasm (Figure 1A). Although many of the molecular details of NPC insertion have been elucidated in recent years (reviewed in [7,8]), the membrane remodeling events leading to pore formation in the nuclear envelope remain incompletely understood.

## 2. NPC Formation

Two distinct modes of NPC formation exist: post-mitotic NPC assembly and interphase NPC assembly [7]. As its name indicates post-mitotic assembly occurs right after mitosis. During mitosis the mitotic spindle apparatus needs to gain access to the chromosomes. Some organisms like the fission yeast (*Schizosaccharomyces pombe*) practice closed mitosis [10], where the spindle microtubules are located in the nucleus or alternatively can reach through the nuclear membrane, allowing mitosis to take place with the nuclear membrane mostly intact. In metazoa, however, the mitotic spindle is located in the cytoplasm while the chromosomes are located in the nucleus, with the nuclear membrane posing as a physical barrier between them. For this reason, metazoa practice open mitosis. During open mitosis, the nuclear membrane, including the NPCs, completely breaks down, allowing the spindle microtubules to interact with the chromosomes. During the mitosis proper, parts of the nuclear membrane and pre-assembled subunits of the NPCs are stored in the cytoplasm [11], ER [12], or other cell components [13,14]. At the end of mitosis, some of the stored NPC subunits become chromatin associated. The nuclear envelope then reassembles, enclosing the chromatin-associated NPC precursors in the nascent membrane as the NPC maturates [15].

In contrast, during nuclear expansion in G1 and G2 phases of the cell cycle, novel NPCs are embedded into the growing, but still intact, nuclear envelope, so as to allow the doubling of the NPC number, which is required for the equal distribution of NPCs in mother and daughter cells [16]. Electron microscopy investigations indicate that interphase pore assembly is initiated from the nucleoplasmic side and proceeds through inside-out evaginations of the INM, which, upon maturation, fuse with a flat ONM (Figure 1B) [9]. This means that the nuclear envelope must be locally remodeled into highly curved membrane structures (also called the NE rim) to allow for close apposition of the INM to the ONM prior to fusion, which is not necessary during post-mitotic assembly. Accordingly, the N-terminal domain of the NPC component nucleoporin 133 (Nup133), which contains a membrane curvature-sensing domain, has been found to be indispensable for interphase but not post-mitotic NPC biogenesis [17].

## 3. Membrane Curvature

**Figure 2:**
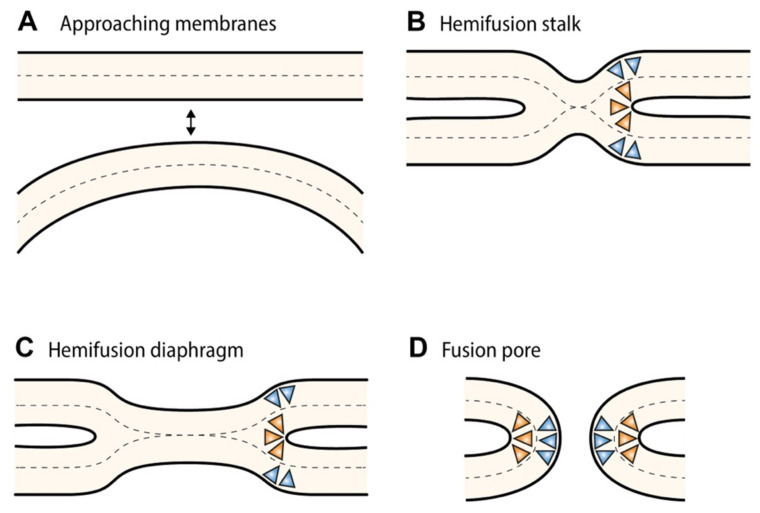
Stalk-pore model of membrane fusion. (**A**) Initial configuration with one membrane approaching the other membrane (or two membranes approaching each other). (**B**) Formation of the (hemifusion) stalk, in which the two proximal (i.e., contacting) monolayers have fused, but not the distal ones. (**C**) Radial expansion of the stalk structure results in the formation of the hemifusion diaphragm. (**D**) Perforation of the bilayer consisting of both distal leaflets leads to the final configuration of the fusion pore. The formation of highly curved fusion intermediates is promoted by the addition of negative-curvature-inducing factors (e.g., cone-shaped lipids, orange triangles) in the proximal (i.e., contacting) leaflets or positive curvature-inducing factors (e.g., amphiphilic helices or inverted cone-shaped lipids; blue triangles) in the distal leaflets. Adapted from [20].

Membranes can be deformed in two directions: they can bulge outwards from the bilayer plane, forming a convex surface (called ‘positive’ curvature), or bend inwards, giving rise to a concave surface (called ‘negative’ curvature). It should be noted, however, that the convention regarding the sign of the curvature is arbitrary [18]. Here, positive curvature refers to bending of the membrane towards the nucleoplasm. Conversely, negative curvature of the membrane promotes its bending towards the luminal (or cytoplasmic) side. From a local perspective, the NE rim is characterized by a complex combination of positive and negative curvature (a so-called ‘saddle-like’ topology), similar to what has been observed at the neck of budding vesicles [19]. During interphase NPC insertion, the dome-shaped INM evagination extends in a direction perpendicular to the bilayer plane [9], which requires a strong negative curvature at most positions in the membrane, whereas a positive curvature strain needs to be imposed at the neck region and, accordingly, to stabilize the pore membrane upon fusion [19] (i.e., stalk-pore model of bilayer fusion; see also Figure 2).

## 4. Proteins and Membrane Curvature

Due to their elastic properties, pure lipid bilayers exhibit resistance to bending deformations [21]. This resistance makes the outward herniation of the INM toward the intermembrane space an energy-demanding process. In order to overcome the energetic barriers associated with generating membrane curvature, cells have evolved a variety of molecular mechanisms to dynamically reshape the membrane, which often involve membrane-protein interactions [22]. For example, asymmetric insertion of amphipathic helices [23] or hydrophobic protein domains (e.g., hairpins) [24] can act as a wedge in only one leaflet and can locally curve the membrane. Alternatively, other protein modules (like the banana-shaped BAR domains [25]) can sculpt membranes by imposing their intrinsically curved shape on the membrane surface through electrostatic interactions. Local protein clustering and self-organization into membrane-spanning protein coats (such as in COPI/II- [26] or clathrin-dependent vesicle formation [27,28]) can further contribute to membrane scaffolding, augmenting the degree of membrane curvature imparted by protein monomers. Of interest, several nucleoporins (i.e., Nup1 [29], Nup53 [30], Nup60 [29] and the Nup133- containing yNup84 complex [17]) and proteins of the reticulon family (like RTN4a and NogoA) [31,32] have been implicated in membrane-remodeling events at the nuclear pore membrane. However, in agreement with the localization of these Nups at the base of the INM protrusion (as demonstrated by the appearance of a density at the corresponding site on electron micrographs [33]), each of these proteins is thought to either induce or stabilize the convex positive surface at the neck region through amphipathic helix insertion. Hence, their concerted action cannot account for the large negative membrane curvature observed during interphase NPC assembly (as noted before by [19]). To fill this explanatory gap, several theories have been proposed. Binding of the integral membrane protein Ndc1 to Nup53 could antagonize the membrane-deformation activity of Nup53, either by oligomerizing into a curved scaffold around the membrane, thereby directly imposing negative curvature, or by fine-tuning Nup53 function through conformational changes [34]. In addition, the Torsin AAA+ ATPase, which primarily resides in the ER and perinuclear space, may be involved in transporting membrane-bending proteins from the ER to the INM. Given that the INM blebs observed in Torsin-deficient HeLa cells are highly similar in shape and dimensions to normal interphase NPC assembly intermediates but lack fusion of the INM to the ONM [35,36], one might speculate that Torsin specifically acts as a trafficking ‘chaperone’ for proteins with fusogenic properties. Alternatively, Torsin could directly participate in INM-ONM fusion, particularly given that it was found that Torsin is able to oligomerize into long helical filaments and induce membrane tubulation in vitro [37]. Another candidate is the endosomal sorting complex required for transport (ESCRT) III together with the AAA+ ATPase Vsp4. Based on the capacity of ESCRT-III to polymerize into a spiral structure lining the neck of budding vesicles [38], it has been hypothesized that ESCRT-III, apart from its role in NPC surveillance [39], might also play a role in interphase NPC assembly by providing the negative curvature strain required for INM evagination [40]. Although evidence for such a role in nuclear pore assembly during mitosis is currently missing, a recent study [41] has shown that ESCRTIII may play a role during NE remodeling during meiosis.

## 5. Lipids and Membrane Curvature

Early studies have demonstrated that changes in lipid composition can support the formation of membrane fusion intermediates and occasionally fusion pores in protein-free lipid bilayers [42]. This suggest that lipids themselves are capable of driving membrane bending. However, the role of membrane lipids in nuclear pore assembly appears to be largely overlooked in the currently published literature. In the following sections of this review, we will discuss how lipid composition and its regulation can contribute to the generation of membrane curvature, with a particular focus on lipid homeostasis at the nuclear pore membrane.

### 5.1. Lipid Shape and Membrane Curvature Are Intrinsically Coupled

In mammalian cells, membranes are composed of a complex mixture of lipid molecules with varying chemical architectures. The most common are (glycero)phospholipids, sphingolipids, and sterols (such as cholesterol) [43]. Membrane lipids can self-assemble into different structures with highly diverse curvatures depending on their intrinsic molecular shapes [44]. Fundamentally, the overall geometry of a lipid is defined by the relative cross-sectional areas of the hydrophilic head group and the hydrophobic fatty acyl chains. Apart from steric size of the head group, the effective head group size also depends on its electric charge [45,46] and hydration radius [47]. For example, charged head groups have a larger effective radius than neutral or zwitterionic head groups due to electrostatic repulsion [45,48]. Change in pH [49] and cation–lipid interactions [46,50] can further modulate the lipid shape. The molecular geometry of the lipid molecule also depends on its acyl chain length and degree of desaturation. Any double bond in the hydrocarbon chain, particularly those in cis configuration, introduces a kink, causing the molecule to adopt a more wedge-shaped conformation, which forces it to occupy more space than its saturated counterpart.

**Figure 3:**
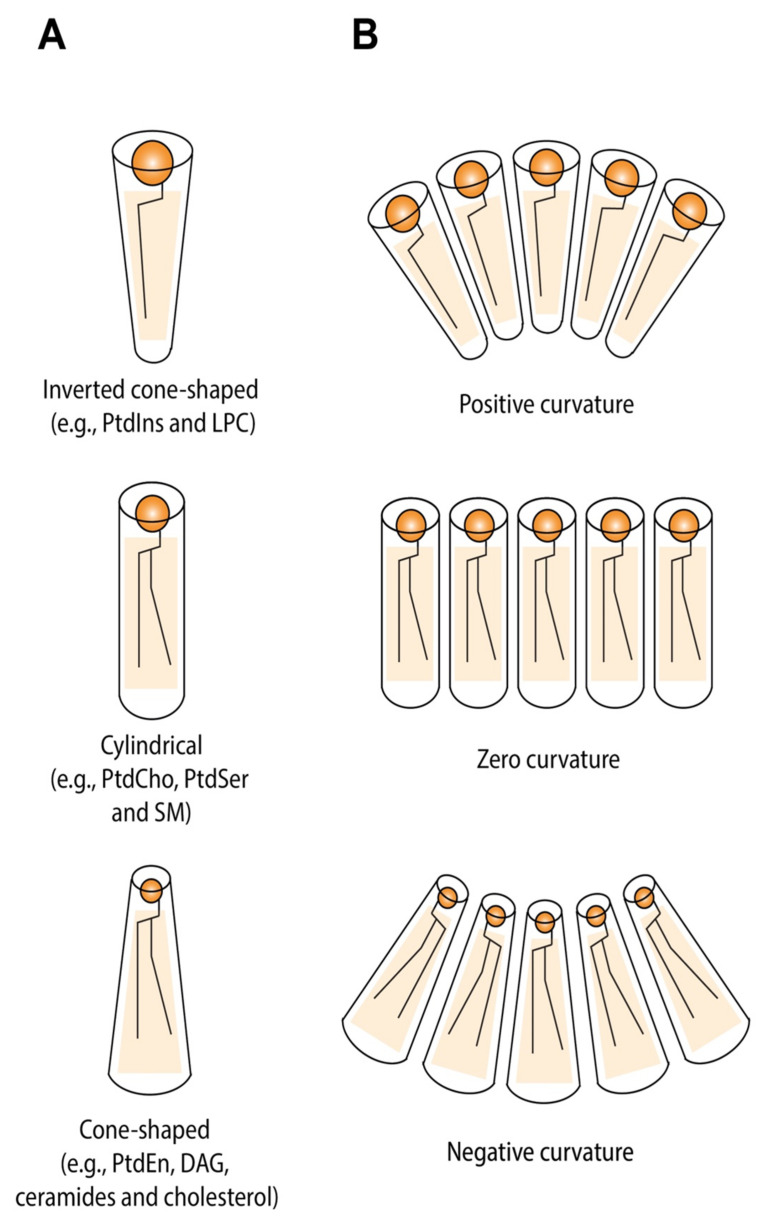
Membrane curvature depends on the shape of the constituent lipid species. (**A**) The molecular geometry of membrane lipids depends on the cross-sectional area of the lipid headgroup versus the three-dimensional volume occupied by the hydrophobic acyl chain. Lipids with a large headgroup are defined as inverted cone-shaped, lipids like PtdCho are roughly cylindrical, while lipids with small headgroup relative to a larger hydrophobic part are cone-shaped. (**B**) Lipid geometry influences membrane curvature. Cone-shaped and inverted cone-shaped lipids self-assemble into monolayers with negative and positive curvature, respectively, while monolayers consisting of cylindrical lipids exhibit zero curvature. PtdIns, phosphoinositol; LPC, lysophosphatidylcholine; PtdCho, phosphatidylcholine; PtdSer, phosphatidylserine; SM, sphingomyelin; PtdEtn, phosphatidylethanolamine; DAG, diacylglycerol.

Based on the relative volumes of the head group and the incorporated acyl chains, membrane lipids can be roughly divided into three basic shapes, each forming monolayers with different spontaneous (or intrinsic) curvatures (Figure 3) [44]: lipids with small head groups relative to a large (often polyunsaturated) fatty acyl domain have a conical shape and bend towards the hydrophobic fatty acyl layer. Examples include phosphatidylethanolamine (PtdEtn), phosphatidic acid (PA), diacylglycerol (DAG), cardiolipin, and cholesterol. Conversely, lipids with bulky, charged head groups and saturated tails like phosphatidylinositol (PtdIns) and gangliosides, or only a single aliphatic tail, like lysophosphatidylcholine (LPC) tend to have an inverted-cone shape and curve spontaneously towards the polar head groups. Phosphatidylcholine (PtdCho), phosphatidylserine (PtdSer), and sphingomyelin (SM), which are characterized by a head group-to-acyl chain ratio roughly equal to 1, have a cylindrical shape and form an essentially flat monolayer. Accordingly, conical, inverted cone, and cylindrical shapes have negative, positive, and zero spontaneous curvatures, respectively.

### 5.2. Transbilayer Lipid Asymmetry Is a Major Driving Force for Membrane Curvature

Under ideal circumstances, a monolayer will curve to achieve the spontaneous curvature dictated by the head-and-tail chemistry of the constituting lipids. However, in a bilayer configuration, monolayers with different bending properties cannot independently adopt their intrinsic curvature without separating from each other, risking the formation of “voids” in the hydrocarbon region. Since hydrophobic voids inside the bilayer would be energetically unsustainable, the bilayer bends to accommodate the asymmetric tension between the monolayers, with the magnitude and sign of the curvature depending on the weighted sum of the spontaneous curvatures of the monolayers (which, in turn, depend on the shape of its constituent lipids) [44]. Owing to this ‘bilayer coupling’, asymmetrical distribution of lipids between monolayers, or local clustering of non-bilayer forming lipids (i.e., cone-shaped and inverted cone-shaped lipids) in nanoscale membrane domains, can drive membrane curvature events (Figure 2). One should take into account, though, that local trans-membrane asymmetry is most effective in inducing curvature when established in a relatively small compartment, such as a vesicle or spatially segregated membrane domain. If not, the effect will average out over a large surface area, thereby rendering the net influence on membrane curvature close to zero [51].

### 5.3. Lipid-Packing Defects Facilitate Insertion of Curvature-Inducing Proteins

Apart from directly affecting the curvature of a membrane, lipid composition can also indirectly shape membranes by facilitating the interaction with proteins with membrane-deforming properties. For instance, bacterial toxins of the cholesterol-dependent cytolysin (CDC) superfamily require the presence of cholesterol in the target membrane for efficient pore formation [52]. Synaptotagmin preferentially inserts in membranes enriched in anionic phospholipids (such as PtdSer) and is able to induce positive curvature, thereby driving the fusion of synaptic vesicles with the plasma membrane [53]. Moreover, cone-shaped lipid molecules, often PtdEtn or lipids with polyunsaturated fatty acyl chains, can reduce lateral packing of lipid head groups, which exposes the hydrophobic membrane core to the surrounding aqueous environment [54]. Such lipid-packing defects can aid the insertion of amphiphatic or hydrophobic motifs in one leaflet of the bilayer [24,55,56], which can then locally deform the membrane depending on the size of the hydrophobic residues: the α-synuclein helix, which contains relatively small residues (i.e., Ala, Thr, Val) prefers shallow defects, whereas amphiphatic lipid packing sensor (ALPS), which harbours more bulky residues (i.e., Leu, Phe), requires deep lipid-packing defects for membrane insertion [57], suggesting that different classes of amphiphatic helices can be specifically targeted to compositionally distinct membrane domains.

Interestingly, curvature can further accentuate hydrophobic defects by increasing the spacing between lipid head groups; accordingly, amphipathic helices and hydrophobic hairpins can be regarded as curvature-sensing motifs, which are recruited to curved membrane sites and further enhance this curvature, in turn facilitating the insertion of additional curvature-sensing and curvature-generating proteins [58,59]. Therefore, by varying lipid-packing density and thus penetration depth, the regulation of lipid composition provides an additional mechanism for modulating membrane curvature.

## 6. Lipid Composition of the Nuclear Envelope: Implications for Membrane Curvature

Early studies of rat and bovine liver nuclei [60,61,62,63] have reported that phospholipids comprise ~65% of the total nuclear membrane lipids, showing a typical phospholipid profile consisting of mostly PtdCho (44–62%) and PtdEtn (12–25%) with lesser amounts of PtdIns (10–15%) and PtdSer (4–9%). In addition, small but significant amounts of SM (3–9%), LPC (1–6%), and cardiolipin (<1–3%) were recovered from the nuclear membrane extracts. Free cholesterol was the most predominant component (6–10% of total lipid) detected among the neutral lipids. Analysis of fatty acid acyl chain composition [56,57,58] revealed high levels of unsaturated fatty acyl groups (48–49%), such as C18:2 (linoleic acid; 8–11%), C20:4 (arachidonic acid; 6–27%), and C22:6 (docosahexaenoic acid; <1–9%), among polyunsaturated fatty acids (PUFAs) and C18:1 (oleic acid; 6–21%) as the principal monounsaturated fatty acid species. C16:0 (palmitic acid; 17–22%) and C18:0 (stearic acid; 26–33%) were the major saturated fatty acid components found in the nuclear membrane. The large variation in the reported levels of fatty (in particular, C20:4 and C22:6) may reflect heterogeneity in the occurrence of lipid peroxidation between studies, given that lower variation was reported with respect to less reactive saturated acyl chains. Accordingly, overall higher PUFA levels were detected in one study [63] that employed antioxidants to minimize endogenous lipid peroxidation.

### 6.1. The Nuclear Envelope Has Low Cholesterol and High Polyunsaturated Fatty Acid Content

Similar to membranes of the ER and cis-Golgi [64], the nuclear envelope is low in cholesterol with a relatively high abundance of lipids with bulky monounsaturated hydrocarbon chains. Because of the ‘kinked’ geometry of unsaturated fatty acids, such membranes are often more fluid and less densely packed (especially at low cholesterol levels) [65,66] than, for instance, the plasma membrane, which, in accordance with its function as a cell-protecting barrier, is enriched in cholesterol and straight, saturated acyl chains. The loose lipid-packing of the nuclear bilayer favours exposure of hydrophobic patches in response to changes in membrane curvature, allowing, for example, the insertion of ALPS-containing Nups (e.g., Nup133 [56]). On the other hand, organelles with more tightly packed membranes (e.g., trans-Golgi and plasma membrane), which are often rich in negatively charged phospholipids, rely on electrostatic interactions with BAR domain-containing proteins for curvature generation [67]. In addition, the relative abundance of flexible PUFAs in the nuclear envelope could potentially reduce the energetic cost of membrane-bending events due to their conformational plasticity. Using in vitro reconstitution assays with liposomes and recombinant proteins, Pinot et al. (2014) have previously shown that PUFAs can facilitate endocytosis and promote membrane fission by the dynamin-endophilin complex [68]. In the same study, it was found that PUFAs disfavoured membrane adsorption of ALPS motifs by promoting the formation of shallow lipid-packing defects, which was even more pronounced in curved membranes. Given that Nups are thought to contribute to early membrane curvature during interphase NPC assembly, one might speculate that prior to Nup binding, local changes in lipid composition are required to facilitate optimal insertion. Interestingly, the degree of membrane fluidity dictates the pore-forming mechanism of the perforin (PFN) and cholesterol-dependent cytolysin (CDC) protein superfamily [52,69]. In the case of PFN, more fluid bilayers composed of lipids with less ordered acyl chains, such as 1,2-dioleoyl-sn-glycero-3-phosphocholine (DOPC) or 1,2-diphytanoyl-sn-glycero-3-phosphocholine (DPhPC), allowed for the rapid insertion of PFN oligomers into the membrane of giant unilamellar vesicles (GUVs), forming small proteolipid pores, which may then mature by the addition of more oligomers into a larger, stable proteinaceous pore. On the other hand, more ordered lipids, like 1-palmitoyl-2-oleoyl-sn-glycero-3- phosphocholine (POPC), minimized the prompt penetration of the β-hairpins of PFN oligomers, leading to the preferential formation of a fully assembled ring-shaped pre-pore complex prior to its insertion into the membrane [69]. Taken together, these findings support the idea that interphase NPC assembly is likely to proceed via the sequential addition of individual NPC components rather than through direct incorporation of a stable pre-assembled NPC (sub)complex (as might be the case for post-mitotic NPC assembly).

### 6.2. The Inner and Outer Nuclear Membrane Are Compositionally Distinct

Although methods for biochemical fractionation of the nuclear envelope have been developed, it still proves difficult to obtain pure ONM and INM fractions in sufficient amounts (in particular, for lipids that occur only in small pools, such as DAG) to allow for lipidomic analysis [70]. Consequently, only few studies have focused on the lipid composition of separated ONM and INM. Among these, one study found that the ganglioside GM1 forms a tight complex with the sodium-calcium exchanger (NCX) in the INM, where it regulates calcium transport from the nucleoplasm to the ER [71], but its presence in the ONM has also been reported [72]. Similarly, its metabolic precursor GD1a was also detected in both INM and ONM [71]. Using GM1-doped GUVs, it was previously shown that asymmetric distribution of GM1 induces membrane tubulation and is associated with the generation of negative spontaneous curvature [73]. Accordingly, other studies found that only small compositional asymmetries with respect to either GM1 or GD1a are capable of driving large changes in membrane curvature [74]. Together, these results suggest a potential role for neuraminidases (also termed sialidases), which convert GD1a into GM1, in locally controlling membrane curvature at the nuclear envelope. In addition, two early studies [75,76], based on filipin-staining of membrane cholesterol, found cholesterol to be either highly enriched or only present in the ONM. Later studies [77,78] also reported the presence of cholesterol together with sphingomyelin in the INM, forming nuclear lipid microdomains (or lipid rafts). Given that interphase NPC assembly is considered to be an asymmetric process, which proceeds through inside-out evagination of the INM [9], one might suspect that this non-uniform distribution of cholesterol is functionally important. As mentioned earlier, cholesterol is characterized by a large negative spontaneous curvature due to its cone-shaped geometry. This property has been associated with its ability to promote several membrane-remodelling events, ranging from exocytotic vesicle scission to viral membrane fusion [79,80]. For example, clathrin-mediated endocytosis of epidermal growth factor receptor and transferrin receptor was impeded in cells treated with the cholesterol-lowering agent β-methyl-cyclodextrin [81]. The observation that, upon cholesterol depletion, flat-coated membranes accumulate [82], suggested that cholesterol is necessary to induce membrane curvature to allow for bud formation and eventually vesicle maturation. Additionally, low membrane cholesterol content increased the lifetime of clathrin-coated pits and prolonged hemifusion events in soluble N-ethylmaleimide-sensitive-factor attachment protein receptor (SNARE)-mediated exocytosis, suggesting that cholesterol is also necessary for the completion steps of fusion [83]. The hypothesis is that cholesterol facilitates membrane fusion by lowering the energetic cost for curved fusion intermediates, such as the hemifusion stalk (see Figure 2) on the way to forming a fusion pore [80]. In order to accomplish this, cholesterol promotes lipid mixing between the proximal (i.e., contacting) leaflets of donor and acceptor membranes in a curvature-dependent manner as shown for unilamellar liposomes [84]. At the same time, target membrane cholesterol was found to prevent the nucleation of leakage pores in liposomes [85,86], which are thought to form either in response to the insertion of fusogenic proteins [87] or due to changes in lipid composition intrinsic to bilayer fusion [86]. Consistent with this view, one might speculate that cholesterol in the ONM is important for stabilizing metastable ONM-INM fusion structures and to preserve the integrity of the nuclear pore membrane.

## 7. Lipid Homeostasis at the Nuclear Envelope: Implications for Membrane Curvature

In order to establish and maintain transmembrane lipid asymmetry, lipid-translocating enzymes (i.e., flippases and floppases) can mediate the directional transfer of lipid species between leaflets. Type IV P-type ATPases (P4-ATPases) are aminophospholipid flippases that move PtdSer and PtdEtn from the extracellular to the cytoplasmic leaflet of the plasma membrane, whereas lipid floppases—members of the ABC (ATP-binding cassette) transporter superfamily—aid movement of phospholipids and other lipid molecules to the extracellular (or luminal) side. The activation of non-specific lipid scramblases can dissipate transbilayer lipid asymmetry by facilitating random flip-flop of phospholipids [88,89,90]. Interleaflet flipping of PtdSer by P4-ATPases has been implicated in membrane deformation and vesicle budding events at different stages of the endocytic and exocytotic pathway in yeast [91]. Additionally, the enhanced translocation of PtdCho, another substrate of P4-Atpases, to the cytoplasmic leaflet was demonstrated to be sufficient to trigger inward PM bending and promote membrane tubulation [92]. Together, these findings suggest that P4-ATPase-mediated phospholipids flipping is able to drive membrane curvature by generating an imbalance in leaflet area, regardless of the class of phospholipid species flipped (Figure 3A). Interestingly, however, no lipid-transferring proteins have yet been identified in the membranes of the nuclear envelope, suggesting that other mechanisms might be involved in causing the dynamic changes in local lipid arrangement at the nuclear pore.

### 7.1. INM Possesses Intrinsic Metabolic Activity and Is Enriched in Cone-Shaped DAG

Using genetically encoded lipid biosensors, Romanauska and Köhler (2018) found that the INM in *Saccharomyces cerevisiae* has a distinct lipid profile from the ONM and ER, enriched in diacylglycerol (DAG), which was found to be directly related to local lipid metabolism at the INM [93]. They demonstrated that a pool of lipid-modifying enzymes, namely Cds1 (CDP-DAG synthase), Dgk1 (DAG-kinase), and Pah1/lipin (phosphatidate phosphatase), localizes to the nucleoplasmic face of the nuclear envelope. Together, these three enzymes control the branching point between lipid synthesis and storage by regulating phosphatidic acid (PA) and DAG levels (Figure 3A): In one pathway branch, PA is dephosphorylated by Pah1/lipin to yield DAG, which can be either further metabolized into triacylglycerols (TAG) and then stored in lipid droplets, or phosphorylated by Dgk1 to regenerate PA. In the other branch, Cds1 catalyses the reaction of PA with CTP to form CDP-diacylglycerol, which can be utilized as a substrate for de novo phospholipid synthesis [94].

Cone-shaped DAG is the membrane lipid with the highest negative spontaneous curvature due to its small polar head group (i.e., a single glycerol moiety) [22]. As a consequence, local enrichment of membrane DAG at the nuclear membrane is expected to drive membrane bending. Accordingly, one study [95] found that the localized depletion of DAG from the cis-Golgi reduced curvature at the NE rim during telophase, which suggested that, apart from ER lamellae, DAG-containing vesicles from the cis-Golgi might also be required for closure of NE gaps during post-mitotic NPC assembly. Consistent with this view, one might speculate that DAG also plays a role in generating membrane curvature required for interphase NPC biogenesis, albeit through an alternative mechanism (i.e., local Pah1/lipin-mediated DAG synthesis). Interestingly, Pah1-deficient cells displayed irregularly shaped nuclei with an expanded nuclear envelope that contained intact nuclear pores [96]. Similar morphological defects were observed for deletion mutants of the fission-yeast Nem1-Spo7 protein phosphatase complex, which regulates Pah1 function, suggesting that Pah1 activity might not be involved in NPC assembly [97]. However, assuming that pah1Δ deletion will shift the balance from lipid storage to the CDP-DAG pathway of phospholipids biosynthesis, any change in local PA/DAG pools at the INM could be obscured by other changes in nuclear membrane composition. For example, PA itself, which has a negative spontaneous curvature, and the PtdCho:PtdEtn ratio can analogously affect membrane bending properties.

### 7.2. Phospholipase C Activity Can Induce Negative Membrane Curvature and Fusion Events

DAG can also be produced by phospholipase C (PLC)-mediated cleavage of PtdCho or PtdIns, dependent on the specificity of the enzyme (i.e., PC-PLC and PI-PLC, respectively). In contrast to Pah1/lipin, PLC has been directly implicated in membrane curvature generation, in manners both dependent [98] and independent [99] of the amount of DAG produced. Both PLC subtypes have been identified in the nucleus [100,101], with PI-PLC activity being specifically reported for the nuclear membrane fraction [102]. PI-PLC is generally considered to play a role in nuclear lipid- dependent signal transduction. Additionally, in a cell-free system derived from fertilized sea urchin eggs, local PI-PLC-dependent DAG production was required for the fusion of PtdIns(4,5)P_2_-rich (pro)nuclear envelope precursor membranes [103], suggesting that nuclear DAG has fusogenic properties. However, since sea urchin pronucleus formation is striking dissimilar from NE assembly in vertebrates, it is unclear how far these conclusions can be generalized to other organisms [104]. Interestingly, Allan et al. (1978) proposed a mechanistic model [105] in which the concerted action of a phospholipase (e.g., PLC) in one leaflet and a diacylglycerol kinase (e.g., Dgk1) in the opposing leaflet can augment DAG asymmetry across the lipid bilayer, thereby driving membrane curvature (Figure 3B): PLC-mediated production of DAG from phospholipids in the outer leaflet is followed by a rapid redistribution of DAG to the inner leaflet, causing a simultaneous contraction of the outer leaflet and expansion of the inner leaflet. The phosphorylation of the accumulated DAG into PA may then further expand the inner leaflet, as the enzymatic conversion would favour the continued influx of DAG from the outer leaflet. Moreover, owing to its phosphate head group, PA is likely to occupy a larger effective membrane area than DAG, which would again contribute to the expansion of the inner leaflet. Based on this model, one could imagine that asymmetrical Dgk1 and PLC activity (at the nucleoplasmic and luminal leaflet of the INM, respectively) could be an important driving force for generating negative curvature strain during NE remodelling events, such as nuclear pore formation.

### 7.3. Sphingomyelinases Can Promote Negative Curvature by Local Ceramide Generation

Analogous to phospholipases, sphingomyelinases (SMases) can drive membrane deformation by converting bilayer-forming sphingomyelin (SM) into phosphocholine and ceramide, the latter of which generally has a negative spontaneous curvature [106]. Cholesterol and sphingolipids cluster together in distinct membrane microdomains, called lipid rafts, which are more ordered and less fluid, i.e., in the liquid-ordered phase, relative to the more liquid-disordered surrounding lipids. Owing to their unique physicochemical properties, lipid rafts have been implicated in a variety of biological processes, including protein segregation, signal transduction and vesicle trafficking (e.g., endocytosis) [107]. The in-situ generation of ceramide through the activation of raft-associated SMases is believed to expand small lipid-raft domains into large ceramide-enriched membrane platforms, which is accompanied by large-scale membrane deformations [108]. For example, in giant liposomes consisting of SOPC (1-stearoyl-2-oleoyl-sn-glycero-3 phosphocholine) and C16:0-SM, SMase-induced ceramide production resulted in the formation of ceramide-rich macrodomains that nucleated vesicle formation [109]. In addition to this mechanism, the transbilayer asymmetry of rapidly diffusing lipids, especially ceramides, can also be established through their entrapment within distinct membrane domains in one leaflet relative to the other. For instance, ceramides display a tendency to cluster together, likely driven by intermolecular hydrogen bonding. As a result, given the smaller lipid area of ceramide, the ceramide-containing leaflet will locally condense, promoting negative (concave) monolayer curvature. Moreover, due to its high affinity for the ordered bilayer phase, ceramide preferably partitions into raft-like domains. In a similar fashion, negatively charged lipids, including PA and fatty acids, can become specifically trapped on one side of the membrane in a pH-dependent manner. As mentioned in Section 6.2, the nuclear envelope contains specialized lipid raft-like microdomains enriched in cholesterol and sphingolipids, which are thought to represent an attachment site for active chromatin during DNA- and RNA synthesis processes [78]. The Cho:SM:PtdCho ratio of these membrane domains is regulated by the activity of neutral sphingomyelinases (e.g., nSMase-1), sphingomyelin synthase, which regenerates sphingomyelin from ceramide and PtdCho, while also producing DAG, and reverse sphingomyelin synthetase, suggesting crosstalk between PtdCho and SM metabolism in regulating the DAG/ceramide ratio at the nuclear envelope [110].

### 7.4. The Sphingomyelinase SMPD4 Localizes to the Nuclear Pore and Interacts with Nucleoporins

Interestingly, recent proteomics screens identified the sphingomyelin phosphodiesterase-4 (SMPD4), also termed neutral sphingomyelinase 3 (nSMase-3) or nuclear envelope transmembrane protein 13 (NET13), at the INM [111] in close association with the NPC [112]. However, a number of other studies have reported presence of SMPD4 at the ONM [113], peripheral ER [114], and/or Golgi apparatus [115], but not at the INM [116]. This discrepancy can be explained by considering that the latter studies involved overexpression of SMPD4. Assuming that SMPD4 localizes at the INM by a diffusion-and-retention mechanism, ectopic expression might alter its subcellular localization by saturating SMPD4 binding sites in the INM, causing it to accumulate in other compartments (i.e., those along its biosynthesis and transport pathway) or in cytoplasmic aggregates. Mutations in SMPD4 were found to cause a developmental disorder characterized by progressive microcephaly, hypomyelination congenital arthrogryposis and early-life mortality [113]. Notably, the autosomal recessive syndrome caused by SMPD4 loss shares several overlapping features with the Galloway –Mowat syndrome (GAMOS), which is associated with mutations in components of the Nup107-160 nuclear pore sub-complex (e.g., Nup133 and Nup107) [117]. Accordingly, proteome-wide pull-down assays have revealed physical interactions between SMPD4 and several NPC components (e.g., Nup93, Nup98, Nup107, Nup133, Nup153, and Nup205) [118]. Taken together, these findings suggest that SMPD4 might play a role in NPC assembly. SMPD4 may do so firstly by recruiting Nups to the nuclear pore membrane via protein–protein interactions, and secondly by generating lipid-packing defects through local ceramide production, allowing the insertion of the individual nucleoporins. The local production of ceramide could also contribute additional negative curvature strain to the INM, which is required to drive evagination during interphase NPC assembly.

### 7.5. Yeast Acetyl CoA Carboxylase (acc1/mtr7) Links Very-Long-Chain Fatty Acid Synthesis to Membrane Curvature at the Nuclear Pore

In their search for mutants defective in nucleocytoplasmic mRNA transport in budding yeast, Schneiter et al. (1996) isolated a temperature-sensitive mutant of the yeast acetyl-CoA carboxylase, acc1^ts^/mtr7, which exhibited severe perturbations of nuclear envelope morphology [119]. Interestingly, this mutant was characterized by the separation of the ONM and INM, inner membrane protuberances and the accumulation of vesicles in the intermembrane space, suggesting a direct link between fatty acid synthesis and the structure of the nuclear envelope. The observed phenotype was strikingly distinct from that exhibited by nucleoporin mutants, such as the grape-like NE-NPC clustering in Nup145, Nup84, and Nup85 mutants and the blister-like NE herniations in Nup116-deficient cells (reviewed by [120]). Acc1/mtr7 catalyzes the carboxylation of acetyl-CoA to generate malonyl-CoA, which serves as a carbon donor for the de novo synthesis of long-chain fatty acids (LCFAs) and their subsequent elongation to very-long-chain fatty acids (VLCFAs), such as C26:0 fatty acyl species [121]. The authors [119] hypothesized that, if the amount of VLCFAs becomes limiting, the 180° bend which connects the ONM and INM at the nuclear pore is destabilized and finally breaks, forming a protuberance upon resealing of the ONM. The C26 fatty acyl chain is incorporated in the ceramide moiety of sphingolipids and GPI (glycosylphosphatidylinositol)-anchored proteins [122,123], both of which are essential for viability in yeast. However, conditional mutants affecting ceramide synthesis and GPI-anchor remodelling pathways did not exhibit the characteristic alterations in nuclear envelope morphology observed in acc1^ts^/mtr7 mutant cells [123]. Mass-spectrometric lipid analysis of nuclear membrane fractions revealed the presence of an inositol-containing C26-substituted glycerophospholipid (C26-PI) with distinct biophysical properties. In contrast to PIs with shorter fatty acyl-chain lengths, C26-PIs significantly lowered the bilayer-to-hexagonal phase transition temperature when added to liposomes composed of dielaidoylphosphatidylethanolamine (DEPE). This observation suggested that C26-PIs can stabilize regions of high negative curvature (similarly to long-chain ceramides), potentially by occupying the ‘void volume’ in the hydrophobic interior of the membrane [123]. Together, these findings suggest that the defect in interphase NPC assembly found in acc1^ts^/mtr7 mutant cells may directly depend on the molecular architecture of the lipid pore membrane, rather than upon the proteinaceous NPC structure assembled therein. However, it cannot be ruled out that an integral membrane protein of the NPC, which might stabilize the ONM-INM interface, has stringent requirements for the lipid environment critical to its function [119].

Three yeast integral membrane proteins, namely Apq12, Brl1, and the closely related Brr6 protein, have also been implicated in lipid homeostasis at the NE membrane [124,125,126,127]. These proteins form a complex together and localize to the INM evaginations of emerging NPC assembly sites, where they are believed to promote the biogenesis of new NPCs. The exact mechanism through which this occurs, however, remains unknown. Loss of expression of either Apq12, Brl1, or Brr6 was associated with aberrant NE morphology and rendered yeast nuclear membranes hypersensitive to changes in temperature (as well as to the membrane-fluidizing agent benzyl alcohol): while mutants were able to sense the temperature shift and modify their membrane lipid composition accordingly, they did so in an overcompensatory manner. The nuclear membranes of mutants accumulated higher levels of monounsaturated versus saturated acids, with overall shorter fatty acyl chain lengths, compared to their wild-type counterparts, resulting in increased membrane fluidity. The authors [124] concluded that Apq12, Brl1, and Brr6 form parts of a homeostatic regulatory system that monitors the need to locally modify the physicochemical properties of the nuclear membrane (e.g., membrane fluidity) in response to environmental perturbations. Accordingly, they hypothesized that the concerted action of these proteins might contribute to creating a local membrane environment that is conducive to dynamic membrane remodelling events, such as nuclear pore formation. In a more recent study [128], however, no obvious changes in the fatty acid composition of the nuclear envelope were observed in Brl1/Brr6 double-knockout cells, nor upon the overexpression of either of both genes. These findings suggest that Brl1 and Brr6 might promote NPC biogenesis through an alternative mechanism, potentially by recruiting or scaffolding Nups during NPC assembly or through yet-to-be-demonstrated direct membrane-remodelling activity.

## 8. Towards a Lipid-Inclusive Model of Nuclear Pore Formation

Despite more than half a century of active research since the discovery of the nuclear-pore complex (NPC) [129], the lipid composition of the nuclear envelope (NE) has been largely ignored in our understanding of nuclear pore formation. This is, arguably, the case for two reasons. Firstly, since the NE is continuous with the ER membrane and lipids can freely diffuse through membrane continuities [130] (such as those at the pore membrane), it is generally assumed that the INM passively receives lipids from the ER. Consistent with this view, the INM is considered a “metabolically silent territory” [93], which resembles the ONM/ER in respect of its lipid composition. Secondly, although biochemical fractionation and isolation methods have been developed to allow for the comparison of outer and inner nuclear membrane composition, such procedures often take very long, such that levels of individual lipid species can easily change due to their conversion into other lipid classes by lipid-modifying enzymes present in the membrane fractions [70], making it difficult, if not impossible, to capture any transient and dynamic changes in lipid composition accompanying nuclear pore formation. However, the finding by Romanauska and Köhler (2018) that the INM is not metabolically inert, but is instead actively synthesizing and modifying its own unique phospholipid constituents, has underlined the potentially underestimated role of lipid composition and metabolism in nuclear pore formation. In support of this role, some authors have argued that proteins alone are not sufficient for explaining the localized extreme curvature required for nuclear membrane fusion during interphase [131]. In addition, others have noted that most of the NPC-related proteins identified so far cannot account for the highly negatively curved membrane surfaces found at the pore membrane [19]. In this review, we have discussed how lipids can participate in membrane curvature generation and how this is required at different stages of nuclear pore formation. Early changes in membrane curvature could locally activate phospholipases and sphingomyelinases, which, due to their enzymatic activity, can further modulate lateral packing density of membrane phospholipids, allowing for the cooperative recruitment of curvature-generating proteins to the nuclear rim. Local synthesis of cone-shaped lipids, such as DAG and long-chain ceramides, can provide the negative curvature required for the budding of the INM into the perinuclear space, while the insertion of ALPS-containing nucleoporins stabilizes the positively curved pore neck region. In addition, membrane helices of integral membrane proteins at the base of the pore may serve as yet-to-be-identified lipid diffusion barriers, which could sub-compartmentalize the pore membrane and spatially restrict the diffusion of lipid species to a small area to allow for more efficient curvature generation. Furthermore, given their intrinsic fusogenic properties, some membrane lipids can reduce the energetic barrier to membrane fusion, while others (such as cholesterol and very-long-chain phosphoinositides) could aid in stabilizing the highly curved membranes at the edge of the open fusion pore. From within such a lipocentric framework, the lipids lining the inner surface of the pore as well as the lipid-modifying enzymes involved in their biosynthesis play important structural roles in the formation of a ‘proteolipidic’ pore. Given that pure lipid pores are typically, intrinsically metastable and short-lived with a lifetime dependent on the frequency of lipid fluctuations [132], proteins are undoubtedly essential (in particular when taking into account the average lifetime of the nuclear pore, i.e., ~40 h [16]). However, such proteins can be best described as pore-enhancing or pore-stabilizing rather than pore-forming elements. They may increase the probability and lifetime of membrane pores [133], e.g., by lowering the activation energy required for pore formation and stabilizing the open pore structure or by preventing pore-resealing through lipid-protein interactions at the pore edge [134]. Changes in the mechanical properties of the nuclear membrane (as reviewed elsewhere [135]), such as membrane tension and lateral compressive stress in the lipid bilayer, might further stabilize the extremely curved membrane configuration at the nuclear rim and contribute to phenomena such as the uniform spacing and the expansion of the nuclear pores [136]. In this context, the nuclear lamina and other factors tethering the INM to chromatin and the cytoskeleton, such as A-type lamins [137] and SUN proteins [135], might play hitherto unexplored roles in nuclear pore formation by dynamically influencing nuclear membrane mechanics (e.g., stiffness).

Future research, as highlighted by this review, should be aimed at exploring the overlooked role of lipids in nuclear pore formation, with a particular focus on how dynamic changes in membrane lipids generate and stabilize the high-curved regions at the pore membrane during interphase. In a similar fashion to Romanauska and Köhler (2018) [93], genetically encoded lipid biosensors can be employed to probe dynamic changes in membrane lipid environment, which can be complemented with correlative fluorescence and electron microscopy to study the accompanying ultrastructural changes in NE and NPC morphology. Additionally, a proximity-dependent labelling proteomics approach (e.g., BioID) [138] could further assist in identifying the local protein and lipid interactome of the nuclear pore complex.
